# Quantifying the impact of different approaches for handling continuous predictors on the performance of a prognostic model

**DOI:** 10.1002/sim.6986

**Published:** 2016-05-18

**Authors:** Gary S. Collins, Emmanuel O. Ogundimu, Jonathan A. Cook, Yannick Le Manach, Douglas G. Altman

**Affiliations:** ^1^ Centre for Statistics in Medicine, Nuffield Department of Orthopaedics, Rheumatology and Musculoskeletal SciencesUniversity of OxfordWindmill RoadOxfordOX3 7LDU.K; ^2^ Departments of Anesthesia and Clinical Epidemiology and BiostatisticsMichael DeGroote School of Medicine, Faculty of Health Sciences, McMaster University and the Perioperative Research Group, Population Health Research InstituteHamiltonCanada

**Keywords:** prognostic modelling, continuous predictors, dichotomisation

## Abstract

Continuous predictors are routinely encountered when developing a prognostic model. Investigators, who are often non‐statisticians, must decide how to handle continuous predictors in their models. Categorising continuous measurements into two or more categories has been widely discredited, yet is still frequently done because of its simplicity, investigator ignorance of the potential impact and of suitable alternatives, or to facilitate model uptake. We examine three broad approaches for handling continuous predictors on the performance of a prognostic model, including various methods of categorising predictors, modelling a linear relationship between the predictor and outcome and modelling a nonlinear relationship using fractional polynomials or restricted cubic splines. We compare the performance (measured by the *c*‐index, calibration and net benefit) of prognostic models built using each approach, evaluating them using separate data from that used to build them. We show that categorising continuous predictors produces models with poor predictive performance and poor clinical usefulness. Categorising continuous predictors is unnecessary, biologically implausible and inefficient and should not be used in prognostic model development. © 2016 The Authors. Statistics in Medicine published by John Wiley & Sons Ltd.

## 1 Introduction

Categorising continuous measurements in regression models has long been regarded as problematic (e.g., biologically implausible particularly when dichotomising), highly inefficient and unnecessary 1, 2, 3, 4, 5. In the context of clinical prediction, investigators developing new prognostic models frequently categorise continuous predictors into two or more categories 6. Categorisation is often carried out with no apparent awareness of its consequences or in a misguided attempt to facilitate model interpretation, use and uptake. However, categorisation causes a loss of information, and therefore reduces the statistical power to identify a relationship between a continuous measurement and patient outcome 7. As studies developing new prognostic models are already generally quite small, it seems unwise to discard any information 6, 8. Despite the numerous cautions and recommendations not to categorise continuous measurements, there have been relatively few quantitative assessments of the impact of the choice of approach for handling continuous measurements on the performance of a prognostic model 1, 9.

Determining the functional form of a variable is an important, yet often overlooked step in modelling 10. It is often insufficient to assume linearity, and categorising continuous variables should be avoided. Alternative approaches that allow more flexibility in the functional form of the association between predictors and outcome should be considered, particularly if they improve model fit and model predictions 11, 12, 13. Two commonly used approaches are fractional polynomials and restricted cubic splines 14, 15. However, few studies have examined how the choice of approach for handling continuous variables affects the performance of a prognostic model. Two single case studies demonstrated that dichotomising continuous predictors resulted in a loss in information and a decrease in the predictive ability of a prediction model 1, 16. However, neither study was exhaustive, and both studies only examined the impact on model performance using the same data that the models were derived from. In a more recent study, Nieboer and colleagues compared the effect of using log transformations, fractional polynomials and restricted cubic splines against retaining continuous predictors as linear on the performance of logistic‐based prediction models, but they did not also examine models that categorised one or more continuous predictors 9.

When developing a new prognostic model, investigators can broadly choose to (i) dichotomise, or more generally categorise, a continuous predictor using one or more cut‐points; (ii) leave the predictor continuous but assume a linear relationship with the outcome; or (iii) leave the predictor continuous but allow a nonlinear association with the outcome, such as by using fractional polynomials or restricted cubic splines. How continuous measurements are included will affect the generalisability and transportability of the model 17. A key test of a prognostic model is to evaluate its performance on an entirely separate data set 18. It is therefore important that the associations are appropriately modelled, to improve the likelihood that the model will predict sufficiently well using data with different case‐mix.

The aim of this article is to quantitatively illustrate the impact of the choice of approach for handling continuous predictors on the apparent performance (based on the development data set) and validation performance (in a separate data set) of a prognostic model.

## 2 Methods

### 2.1 Study data: The Health Improvement Network

The Health Improvement Network (THIN) is a large database of anonymised electronic primary care records collected at general practice surgeries around the United Kingdom (England, Scotland, Wales and Northern Ireland). The THIN database contains medical records on approximately 4% of the United Kingdom population. Clinical information from over 2 million individuals (from 364 general practices) registered between June 1994 and June 2008 form the data set. The data have previously been used in the external validation of a number of risk prediction models, including those considered in this study 19, 20, 21, 22, 23, 24, 25, 26. There are some missing data for the predictors of systolic blood pressure, body mass index and cholesterol. For simplicity and convenience, we have used an imputed data set used in published external validation studies (details on the imputation strategy are reported elsewhere 20, 27).

### 2.2 Prognostic models

We used Cox regression to develop prognostic models that predict the 10‐year risk of cardiovascular disease and 10‐year risk of hip fracture. We split the THIN database geographically by pulling out two cohorts: general practices from England and general practices from Scotland. Data from the England cohort were used to develop the models, whilst data from Scotland were used to validate the model. Using data for 1,803,778 patients (men and women) from England (with 80,880 outcome events) to develop cardiovascular prognostic models. Model performance was evaluated on 110,934 patients from Scotland (with 4688 outcome events). Similarly, 980,465 women (with 7721 outcome events) in the THIN database from England were used to develop hip fracture models. The model performance was evaluated on data from 61,563 women from Scotland (with 565 outcome events). All data come from the same underlying computer system used to collect the patient level data, and thus splitting this large database geographically is a weak form of external validation (sometimes referred to as narrow validation), although given the health systems in the two countries are ultimately the same the case‐mix is not dissimilar. In this instance, the validation is closer to an assessment of reproducibility than transportability 18.

For convenience and simplicity, the models were developed using a subset of variables that were chosen from the total list of predictors contained in the QRISK2 28 and QFracture 29 risk prediction models. The cardiovascular disease models used age (continuous), sex (binary), family history of cardiovascular disease (binary), serum cholesterol (continuous), systolic blood pressure (continuous), body mass index (continuous) and treated hypertension (binary). The hip fracture models used age (continuous), body mass index (continuous), Townsend score (categorical), diagnosis of asthma (binary) and prescription of tricyclic antidepressants (binary). Table 1 shows the distribution of the model variables in the THIN data set for both outcomes.

**Table 1:** Characteristics of the individuals in the THIN data set, used as predictors in the developed prognostic models; SD: standard deviation.

Variable	Cardiovascular disease		Hip fracture	
	Development (England) (*n* = 1,803,778)	Validation (Scotland) (*n* = 110,934)	Development (England) (*n* = 980,465)	Validation (Scotland) (*n* = 61,563)
Mean age in years (SD)	48.6 (14.1)	48.9 (14.0)	50.8 (15.3)	50.1 (14.9)
Men	922,913 (51.2%)	62,321 (56.2%)	—	—
Family history of cardiovascular disease	74,668 (4.1%)	4,268 (3.8%)	—	—
Mean serum cholesterol (SD)	5.5 (1.2)	5.5 (1.2)	—	—
Mean systolic blood pressure (SD)	131.8 (20.3)	131.6 (20.2)	—	—
Mean body mass index (SD)	26.3 (4.4)	26.3 (4.5)	26.1 (4.9)	26.5 (5.1)
Treated for hypertension	96,634 (5.4%)	6,223 (5.6%)	—	—
Diagnosis of asthma	—	—	84,279 (8.6%)	5,207 (8.5%)
History of falls	—	—	26,124 (2.7%)	1,016 (1.7%)
Prescription of tricyclic antidepressants	—	—	51,849 (5.3%)	3,528 (5.7%)

### 2.3 Resampling strategy

A resampling study was performed to examine the impact of different strategies for handling continuous predictors in the development of prognostic models on the performance of the model when evaluated in a separate data set.

Two hundred samples were randomly drawn (with replacement) from the THIN data set so that the number of events in each sample was fixed at 25, 50, 100 and 2000. Individuals in each sample were chosen by sampling a constant fraction of those who experienced the event and those who did not, according to the overall proportion of events in the THIN data set, for the corresponding prognostic model outcome (cardiovascular disease or hip fracture). Models were developed for each sample as described in Sections 2.2 and 2.4. Model performance (Section 2.5) was evaluated on the same data used to derive the model, to give the apparent performance, and on separate data, for validation performance (Section 2.1).

### 2.4 Approaches for handling continuous predictors

We considered three broad approaches for handling continuous predictors:

1. Categorise each continuous predictor into equally sized groups, using the median value of the predictor to form two groups, the tertile values to form three groups, the quartile values to form four groups or the quintile values to form five groups. We further categorised the age predictor by grouping individuals into 5‐year or 10‐year age intervals. We also categorised the continuous predictors based on ‘optimal’ cut‐points, using a cut‐point that minimised the *P*‐value from the logrank test for over 80% of the observations (the lower and upper 10% of observations removed) 30.
2. Model each continuous predictor by assuming that it has a linear relationship with the outcome.
3. Model each continuous predictor by assuming that it has a non‐linear relationship with the outcome, using fractional polynomials 14 and restricted cubic splines 15. Fractional polynomials are a set of flexible power transformations that describe the relationship between a continuous predictor and the outcome. Fractional polynomials of degree one (FP1) and two (FP2) are defined as

$$FP1\left(x\right)=\beta_{1}x^{p}\text{,}$$

$$FP2\left(x\right)=\left\{\begin{array}{cc} \beta_{1}x^{p_{1}}+\beta_{2}x^{p_{2}}, & p_{1}\neq p_{2}\\ \beta_{1}x^{p_{1}}+\beta_{2}x^{p_{2}}\,\ln x, & p_{1}=p_{2}=p \end{array}\right.$$

where the powers *p*, *p*_1_, *p*_2_ ∈ *S* = {−2, − 1, − 0.5, 0, 0.5, 1, 2, 3} and *x* ^0^ = ln *x*. We used the multivariable fractional polynomial (MFP) procedure in R to model potentially nonlinear effects while performing variable selection 31. MFP formally tests for deviations from linearity using fractional polynomials. It incorporates a closed test procedure that preserves the ‘familywise’ nominal significance level. The default FP2 with significance level of 0.05 was chosen to build the prognostic models. The restricted cubic splines method places knots along the predictor value range and between two adjacent knots, where the association between the predictor and the outcome is modelled using a cubic polynomial. Beyond the outer two knots, the relationship between the predictor and outcome is modelled as a linear association. Three to five knots are often sufficient to model the complex associations that are usually based on the percentiles of the predictor values. We used the rcs function in the rms package in R 32. Prior to conducting the simulations, preliminary analyses suggested that four degrees of freedom should be used to model each continuous predictor with fractional polynomials (i.e., FP2 models) and that three knots should be used in the restricted cubic splines approach. The models with the highest degrees of freedom (df) were those categorising the continuous predictors into fifths (CVD: df = 19, Hip fracture: df = 11), model using fractional polynomials also had a maximum potential degrees of freedom also of 19 and 11 for the CVD and hip fracture models respectively. The fewest degrees of freedom were used by the models that assumed a linear relationship with the outcome, those dichotomising (CVD: df = 7; Hip fracture: df = 5), see Supplementary Table 1.

**Figure 1:**
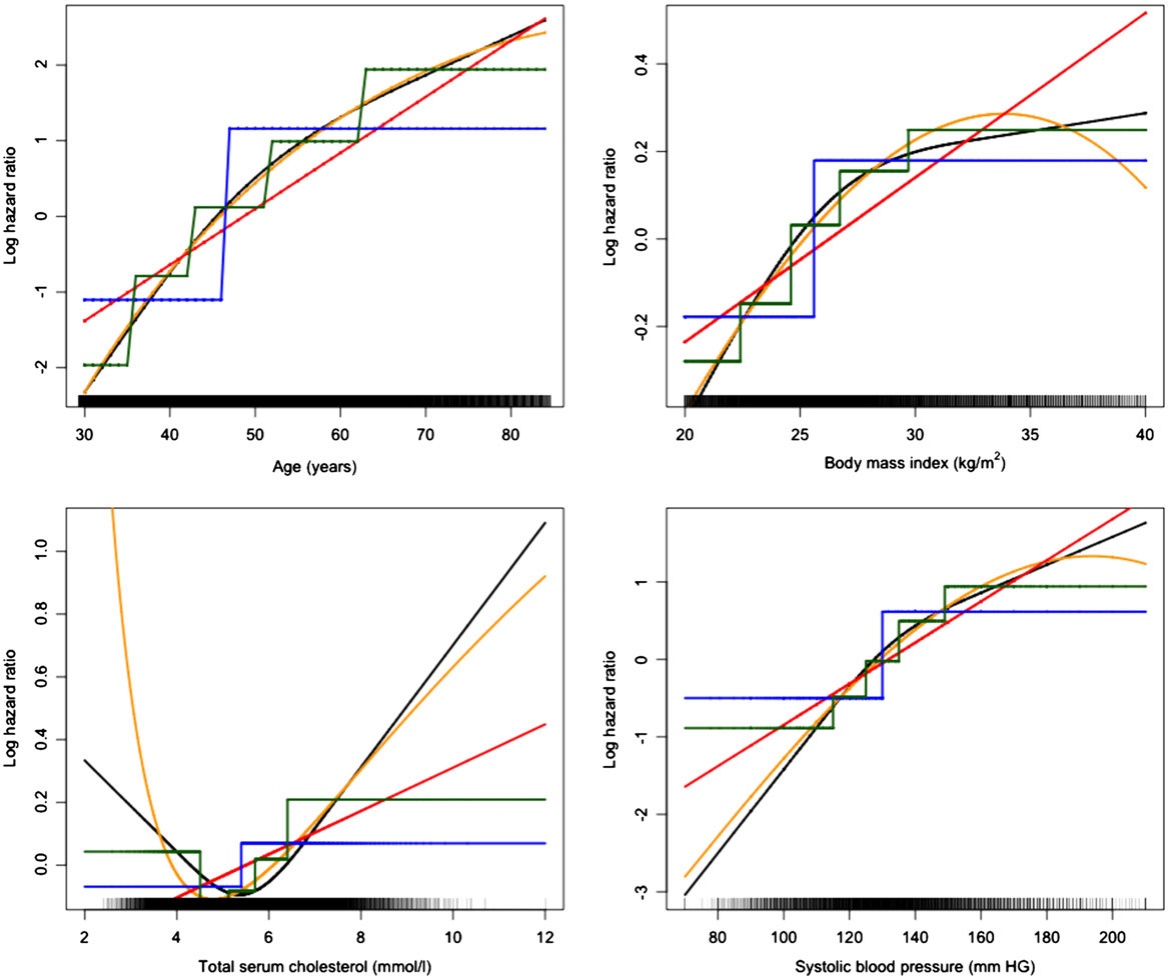
Continuous predictors from the THIN development data set. Models developed to predict cardiovascular disease using the (orange) fractional polynomial approach with four degrees of freedom, (black) restricted cubic spline approach using three knots, (blue) dichotomising at the median predictor value approach, (green) categorising into five equally sized groups approach and (red) linear approach.

Figure 1 shows how the relationship between the continuous predictors (age, systolic blood pressure, body mass index and total serum cholesterol) and cardiovascular disease when estimated, assuming linearity, nonlinearity (fractional polynomials and restricted cubic splines) and using categorisation (Supplementary Figure 1 shows the corresponding relationship for continuous age and body mass index with hip fracture). Age is one of the main risk factors for many diseases, including cardiovascular disease 33 and hip fractures 34; we therefore examined models whereby all continuous predictors apart from age were assumed linear, whilst age was included using the approaches described above.

All of the continuous predictors in the development and validation data sets were centred and scaled before modelling. Scaling and centring reduce the chance of numerical underflow or overflow, which can cause inaccuracies and other problems in model estimation 13. The values used for transformation were obtained from the development data set and were also applied to the validation data set.

### 2.5 Performance measures

Model performance was assessed in terms of discrimination, calibration, overall model performance and clinical utility. Discrimination is the ability of a prognostic model to differentiate between people with different outcomes, such that those without the outcome have a lower predicted risk than those with the outcome. The survival models in this study were based on the time‐to‐event. Discrimination was thus evaluated using Harrell's *c*‐index, which is a generalisation of the area under the receiver operating characteristic curve for binary outcomes (e.g. logistic regression) 35, 36.

We graphically examined the calibration of the models at a single time point, 10 years, using the val.surv function in the rms library in R. For each random sample, hazard regression with linear splines was used to relate the predicted probabilities from the models at 10 years to the observed event times (and censoring indicators). The actual event probability at 10 years was estimated as a function of the estimate event probability at 10 years. We investigated the influence of the approach used to handle continuous predictors on calibration by overlaying plots of observed outcomes against predicted probabilities for each of the 200 random samples.

We evaluated the clinical usefulness, or net benefit, of the models using decision curve analysis 37. Net benefit is the difference between the number of true‐positive results and the number of false‐positive results, weighted by a factor that gives the cost of a false‐positive relative to a false‐negative result 38, and is defined as:

$$Net\;\text{Benefit}=\frac{\text{True Positives}}{n}-\frac{\text{False positives}}{n}\left(\frac{p_{t}}{1-p_{t}}\right)\text{.}$$

The numbers of true and false positives were estimated using the Kaplan–Meier estimates of the percentage surviving at 10 years among those with calculated risks greater than the threshold probability. *n* is the total number of people. *p_t_* is the threshold on the probability scale that defines high risk and is used to weight false positives to false negative results. To calculate the net benefit for survival time data subject to censoring 39, we defined *x* = 1 if an individual had a predicted probability from the model ≥ *p_t_* (the threshold probability), and *x* = 0 otherwise. S(t) is the Kaplan–Meier survival probability at time t (t = 10 years) and *n* is the number of individuals in the data set. The number of true positives is given by [1 − (S(t) | *x* = 1)] × P(*x* = 1) × *n* and the number of false positives by (S(t) | *x* = 1) × P(*x* = 1) × *n*. A decision curve was produced by plotting across a range of *p_t_* values.

## 3 Results

Tables 2 and 3 show the mean (standard deviation) of the *c*‐index over the 200 samples (each containing 25, 50, 100 and 2000 events) for the models produced by each approach that predict the hip fracture and cardiovascular outcomes, respectively. For the hip fracture models, there was a large difference of 0.1 between the mean *c*‐index produced by the approaches that did not categorise the continuous predictors and the approach that dichotomised the continuous predictors at the median. The same *c*‐index difference was observed in the apparent performance (i.e. the development performance) and the geographical validation performance. A similar pattern was observed for other performance measures including the D‐statistic 40, *R* ^2^ 41 and Brier score (see Supplementary Tables 2–7). The differences in all the measures (*c*‐index, D‐statistic, *R* ^2^ and Brier score 42) all favoured the approaches that kept the variables continuous. The approaches that assumed a linear relationship between the predictor and outcome and the approaches that used either fractional polynomials or restricted cubic splines had similar results for all of the performance measures. The variability (as reflected in the SD) in the four model performance metrics was small relative to the mean value for all of the methods of handling continuous predictors.

**Table 2:** c‐index of the hip fracture prognostic models varying the development sample size (200 simulations) [mean (SD)] df: degrees of freedom; SD: standard deviation.

	25 events (development)		50 events (development)		100 events (development)		2000 events (development)	
	Apparent performance	Validation	Apparent performance	Validation	Apparent performance	Validation	Apparent performance	Validation
Linear (all continuous)	0.9017 (0.0289)	0.8678 (0.0335)	0.9027 (0.0209)	0.8828 (0.0122)	0.8993 (0.0148)	0.8860 (0.0022)	0.8979 0.0032)	0.8880 (0.0002)
Age (only) dichotomised at the median	0.8110 (0.0345)	0.7791 (0.0290)	0.8067 (0.0258)	0.7922 (0.0097)	0.8012 (0.0195)	0.7951 (0.0059)	0.7987 (0.0038)	0.7983 (0.0007)
Dichotomised all continuous predictors at the median	0.7991 (0.0318)	0.7654 (0.0265)	0.7938 (0.0223)	0.7780 (0.0131)	0.7872 (0.0184)	0.7820 (0.0067)	0.7846 (0.0038)	0.7864 (0.0015)
Dichotomised all continuous predictors at the optimal	0.8573 (0.0389)	0.7631 (0.0626)	0.8401 (0.0312)	0.7798 (0.0296)	0.8261 (0.0266)	0.7818 (0.0249)	0.7704 (0.0065)	0.7722 (0.0055)
Categorised into 5‐year age categories	0.9059 (0.0241)	0.8554 (0.0201)	0.9011 (0.0194)	0.8705 (0.0100)	0.8946 (0.0142)	0.8780 (0.0048)	0.8907 (0.0031)	0.8847 (0.0004)
Categorised into 10‐year age categories	0.8885 (0.0279)	0.8520 (0.0230)	0.8859 (0.0211)	0.8659 (0.0103)	0.8811 (0.0149)	0.8708 (0.0036)	0.8797 (0.0031)	0.8741 (0.0003)
Categorised all continuous predictors into thirds	0.8563 (0.0295)	0.8218 (0.0210)	0.8558 (0.0215)	0.8349 (0.0103)	0.8491 (0.0163)	0.8403 (0.0050)	0.8426 (0.0033)	0.8394 (0.0009)
Categorised all continuous predictors into fourths	0.8831 (0.0257)	0.8408 (0.0216)	0.8784 (0.0202)	0.8541 (0.0088)	0.8726 (0.0143)	0.8588 (0.0045)	0.8688 (0.0030)	0.8634 (0.0005)
Categorised all continuous predictors into fifths	0.8980 (0.0259)	0.8485 (0.0198)	0.8902 (0.0194)	0.8639 (0.0091)	0.8839 (0.0136)	0.8699 (0.0039)	0.8800 (0.0032)	0.8752 (0.0004)
Age (only) categorised into thirds	0.8584 (0.0302)	0.8281 (0.0236)	0.8586 (0.0223)	0.8411 (0.0094)	0.8529 (0.0161)	0.8453 (0.0040)	0.8474 (0.0032)	0.8432 (0.0006)
Age (only) categorised into fourths	0.8806 (0.0266)	0.8462 (0.0221)	0.8776 (0.0214)	0.8583 (0.0079)	0.8734 (0.0145)	0.8615 (0.0036)	0.8706 (0.0030)	0.8640 (0.0004)
Age (only) categorised into fifths	0.8917 (0.0272)	0.8559 (0.0190)	0.8874 (0.0201)	0.8680 (0.0084)	0.8830 (0.0139)	0.8724 (0.0032)	0.8810 (0.0031)	0.8755 (0.0004)
Fractional polynomials [df = 4]	0.9023 (0.0288)	0.8666 (0.0344)	0.9030 (0.0210)	0.8827 (0.0123)	0.8997 (0.0148)	0.8854 (0.0050)	0.8985 (0.0032)	0.8885 (0.0003)
Fractional polynomials of age only [df = 4]	0.9020 (0.0288)	0.8677 (0.0335)	0.9027 (0.0209)	0.8828 (0.0122)	0.8994 (0.0147)	0.8860 (0.0022)	0.8979 (0.0031)	0.8879 (0.0003)
Restricted cubic splines [3 knots]	0.9041 (0.0292)	0.8656 (0.0324)	0.9047 (0.0203)	0.8821 (0.0121)	0.9002 (0.0146)	0.8859 (0.0024)	0.8985 (0.0032)	0.8882 (0.0002)
Restricted cubic splines of age only [3 knots]	0.9023 (0.0288)	0.8681 (0.0314)	0.9031 (0.0208)	0.8826 (0.0121)	0.8994 (0.0147)	0.8859 (0.0022)	0.8979 (0.0032)	0.8879 (0.0002)

**Table 3:** c‐index of the cardiovascular disease prognostic models varying the development sample size (200 simulations) [mean (SD)] df: degrees of freedom; SD: standard deviation.

	25 events (development)		50 events (development)		100 events (development)		2000 events (development)	
	Apparent performance	Validation	Apparent performance	Validation	Apparent performance	Validation	Apparent performance	Validation
Linear (all continuous)	0.8361(0.0362)	0.7920(0.0197)	0.8211 (0.0263)	0.8043 (0.0096)	0.8183 (0.0187)	0.8095 (0.0036)	0.8153 (0.0039)	0.8141 (0.0004)
Age (only) dichotomised at the median	0.7957 (0.0371)	0.7393 (0.0210)	0.7805 (0.0291)	0.7535 (0.0138)	0.7732 (0.0194)	0.7631 (0.0058)	0.7690 (0.0047)	0.7706 (0.0006)
Dichotomised all continuous predictors at the median	0.7910 (0.0367)	0.7302 (0.0193)	0.7725 (0.0275)	0.7447 (0.0130)	0.7653 (0.0202)	0.7525 (0.0071)	0.7614 (0.0045)	0.7604 (0.0006)
Dichotomised all continuous predictors at the optimal	0.8379 (0.0348)	0.7237 (0.0315)	0.8103 (0.0300)	0.7439 (0.0236)	0.7948 (0.0212)	0.7591 (0.0141)	0.7745 (0.0043)	0.7739 (0.0025)
Categorised into 5‐year age categories	0.8501 (0.0336)	0.7481 (0.0763)	0.8286 (0.0253)	0.7868 (0.0130)	0.8193 (0.0185)	0.8007 (0.0054)	0.8125 (0.0040)	0.8109 (0.0004)
Categorised into 10‐year age categories	0.8327 (0.0359)	0.7690 (0.0219)	0.8160 (0.0272)	0.7888 (0.0106)	0.8101 (0.0192)	0.7982 (0.0045)	0.8063 (0.0042)	0.8046 (0.0004)
Categorised all continuous predictors into thirds	0.8281 (0.0353)	0.7479 (0.0234)	0.8080 (0.0261)	0.7678 (0.0118)	0.7990 (0.0184)	0.7788 (0.0061)	0.7908 (0.0043)	0.7877 (0.0006)
Categorised all continuous predictors into fourths	0.8486 (0.0331)	0.7504 (0.0220)	0.8240 (0.0254)	0.7736 (0.0132)	0.8121 (0.0187)	0.7880 (0.0060)	0.8018 (0.0040)	0.7999 (0.0005)
Categorised all continuous predictors into fifths	0.8636 (0.0295)	0.7418 (0.0258)	0.8347 (0.0251)	0.7732 (0.0134)	0.8205 (0.0182)	0.7903 (0.0070)	0.8079 (0.0039)	0.8061 (0.0006)
Age (only) categorised into thirds	0.8178 (0.0370)	0.7611 (0.0206)	0.8031 (0.0268)	0.7763 (0.0116)	0.7976 (0.0184)	0.7850 (0.0046)	0.7929 (0.0043)	0.7906 (0.0005)
Age (only) categorised into fourths	0.8296 (0.0355)	0.7682 (0.0206)	0.8129 (0.0260)	0.7846 (0.0120)	0.8065 (0.0183)	0.7946 (0.0044)	0.8019 (0.0040)	0.8006 (0.0004)
Age (only) categorised into fifths	0.8344 (0.0340)	0.7698 (0.0207)	0.8188 (0.0260)	0.7886 (0.0105)	0.8117 (0.0184)	0.7989 (0.0043)	0.8068 (0.0040)	0.8058 (0.0004)
Fractional polynomials [df = 4]	0.8349 (0.0364)	0.7810 (0.0405)	0.8230 (0.0256)	0.7973 (0.0453)	0.8184 (0.0191)	0.8078 (0.0048)	0.8168 (0.0039)	0.8155 (0.0005)
Fractional polynomials of age only [df = 4]	0.8336 (0.0363)	0.7857 (0.0213)	0.8218 (0.0261)	0.8013 (0.0099)	0.8179 (0.0187)	0.8084 (0.0037)	0.8149 (0.0039)	0.8136 (0.0004)
Restricted cubic splines [3 knots]	0.8455 (0.0334)	0.7708 (0.0279)	0.8286 (0.0267)	0.7960 (0.0123)	0.8217 (0.0187)	0.8071 (0.0052)	0.8167 (0.0039)	0.8155 (0.0005)
Restricted cubic splines of age only [3 knots]	0.8350 (0.0357)	0.7812 (0.0250)	0.8227 (0.0263)	0.7999 (0.0103)	0.8178 (0.0186)	0.8077 (0.0042)	0.8148 (0.0040)	0.8134 (0.0004)

Similar patterns in model performance were observed in the cardiovascular models. For example, a difference of 0.055 in the *c*‐index between the approaches that assumed a linear or nonlinear relationship between the predictor and outcome and the approach that dichotomised at the median predictor value was observed in both the apparent performance and geographical validation.

**Figure 2:**
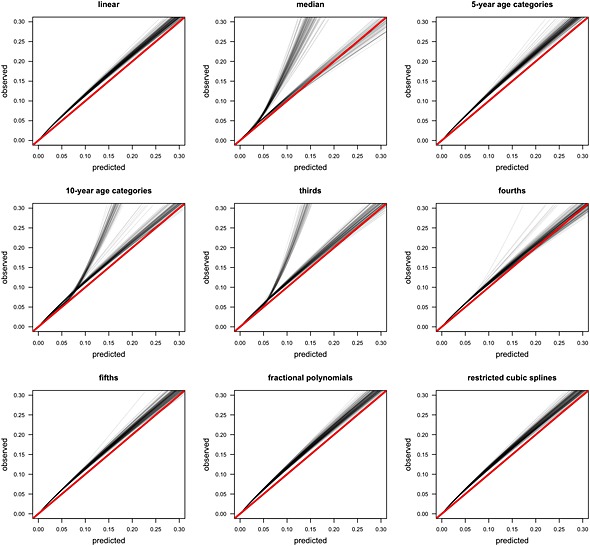
Calibration plots of cardiovascular disease risk in the validation cohort (2000 events).

Figure 2 shows the geographical validation calibration plots for the cardiovascular models. The models using fractional polynomials or restricted cubic splines were better calibrated than the models produced by other approaches, including the models that assumed a linear relationship between the predictor and outcome. A similar pattern was observed in the hip fracture models, although there was more similarity between the linear and nonlinear approaches (see Supplemental Figure 7).

**Figure 3:**
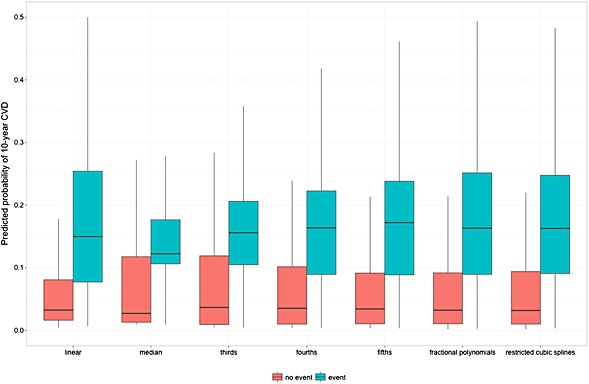
Boxplot of the predicted cardiovascular disease risks in the validation cohort (2000 events).

Figure 3 shows the distribution of the predicted 10‐year cardiovascular risk for a subset of the approaches. The approaches that kept the measurements continuous (linear, fractional polynomial and restricted cubic splines) showed similar predicted risk spreads. The approaches that categorised the continuous predictors showed a noticeably wide predicted risk spread in those who did not experience the outcome, which was widest when the predictor was dichotomised, and showed a noticeable narrow spread for those who did experience the outcome. As the number of categories increases the spread of predicted risk in those who did not have an event decreases, whilst the spread of predicted risk in those who did have an event increases.

Models developed with smaller sample sizes (25, 50 and 100 events) showed higher performance measure values when they were evaluated using the development data set than the models developed with 2000 events. This pattern was observed for both the hip fracture and cardiovascular disease models, regardless of the how continuous predictors were included in the models. In contrast, lower values were observed in the geographical validation of models developed with smaller sample sizes than the models developed with 2000 events, suggesting overfitting. Similarly, models developed with fewer events showed worse calibration on the geographical validation data than the models developed with 2000 events. The models developed by categorising continuous predictors showed the greatest variability in performance.

Table 4 shows the clinical consequences of the different strategies for handling the continuous predictors in the cardiovascular models, using dichotomising at the median predictor value as the reference method. Similar findings were observed in the hip fracture models. Over a range of probability thresholds (e.g. 0.09 to 0.2), an additional net 5 to 10 cardiovascular disease cases per 1000 were found during geographical validation if models that implemented fractional polynomials or restricted cubic splines were used, rather than models that dichotomised all of the continuous predictors at the median without conducting any unnecessary treatment. For models that categorised continuous predictors, more additional cases per 1000 found as the number of categories increases. The models that used fractional polynomials or restricted cubic splines, or that assumed a linear relationship between the predictor and outcome all showed a higher net benefit, over a range of thresholds (0.09 to 0.2), than the categorising approaches (see Supplementary Figure 9).

**Table 4:** Range of additional net cases per 1000 found when using each approach for handling continuous predictors, compared with categorising all of the continuous predictors at the median. Models developed using 2000 outcome events. Range of thresholds 0.09 to 0.2 for cardiovascular disease and 0.01 to 0.05 for hip fracture.

Model	Cardiovascular disease		Hip fracture	
	Development	Validation	Development	Validation
Linear	6 to 11	5 to 10	4 to 6	5 to 7
Fractional polynomials	7 to 11	6 to 10	4 to 6	5 to 7
Restricted cubic splines	7 to 11	6 to 10	4 to 6	5 to 7
5‐year age categories	6 to 10	6 to 9	4 to 6	4 to 7
10‐year age categories	6 to 10	5 to 9	2 to 5	3 to 6
Thirds	2 to 6	1 to 5	1 to 3	1 to 3
Fourths	4 to 8	3 to 7	1 to 4	2 to 5
Fifths	5 to 9	4 to 8	2 to 5	3 to 6

## 4 Discussion

We examined the impact of the choice of approach for handling continuous predictors when developing a prognostic model and evaluating it on a separate data set. We developed models using data sets of varying size to illustrate the influence of sample size. The predictive ability of the models was evaluated on a large geographical validation data set, using performance measures that have been recommended for evaluation and reporting 43, 44.

We have demonstrated that categorising continuous predictors, particularly dichotomising at the median value of the predictor, produces models that have substantially weaker predictive performance than models produced with alternative approaches that retain the predictor on a continuous scale 1. It is not surprising that categorising continuous predictors leads to poor models, as it forces an unrealistic, biologically implausible and ultimately incorrect (step) relationship onto the predictor and discard information. It may appear to be sensible to use two or more quantiles (or similar apparently meaningful cut‐points, such as a particular age) to categorise an outcome into three or more groups, but this approach does not reflect the actual predictor–outcome relationship. Individuals whose predictor values are similar but are either side of a cut‐point are assigned different levels of risk, which has clear implications on how the model will be used in clinical practice. The fewer the cut‐points, the larger the difference in risk between two individuals with similar predictor values immediately either side of a cut‐point. We observed only small differences between the methods that retained the variables as continuous (assuming linearity or nonlinearity). Larger differences would be expected if the relationship between a continuous variable and the outcome were markedly nonlinear.

Continuous predictors are often categorised as the approach is intuitive, simple to implement and researchers may expect it to improve model use and uptake. However, categorising comes at a substantial cost to the predictive performance. We believe that this cost is detrimental and counterproductive, as the resulting models have weak predictive accuracy and are unpopular for clinical use. If ease of use is required, we recommend leaving predictors as continuous during modelling and instead simplifying the final model, using a points system to allow finer calculation of an individual's risk 45, 46, 47.

We focused on exploring the differences in the predictive performance of models produced by each approach for handling continuous predictors. Focusing on a fixed and small number of variables, we circumvented issues around variable selection procedures for which we anticipate the differences in performance to be more marked. Using restricted cubic splines on very small data sets may produce wiggly functions, and fractional polynomials can result in poorly estimated tails. Both problems lead to unstable models that have poor predictive ability when evaluated on separate data in a geographical validation. We observed very little difference in model performance when using either fractional polynomials or restricted cubic splines. Similarly, when a data set is small, the centile values for dichotomising or categorising continuous predictor values may vary considerably from sample to sample, again leading to unstable models and poor predictive performance. We did, however, also examine the influence of smaller sample sizes on model performance and observed greater variability in model performance in small samples with few outcome events than in larger samples. Furthermore, the apparent performance on smaller data sets was higher compared to using larger data set (which decreased as sample size increased), but at the expense of poorer performance in the geographical validation, where recent guidance suggests that a minimum of 100 events are required 48.

Systematic reviews have highlighted numerous methodological flaws, including small sample size, missing data and inappropriate statistical analyses in studies that describe the development or validation of prognostic models 6, 8, 49, 50, 51, 52. Contributing to why many studies have methodological shortcomings is because it is generally easy to create a (poorly performing) prognostic model. A model that has been developed using poor methods is likely to produce overoptimistic and misleading performance. As noted by Andrew Vickers, ‘*we have a data set, and a statistical package, and add the former to the latter, hit a few buttons and voila, we have another paper*’ 53. It is therefore both unsurprising and fortunate that most prognostic models never get used. However, some of these poorly performing or suboptimal prognostic models will undoubtedly be used, which may have undesirable clinical consequences. We have clearly demonstrated that categorising continuous predictor outcomes is an inadequate approach. Investigators who wish to develop a new prognostic model for use on patients that is fit for purpose should follow the extensive methodological guidance on model building that discourages categorisation and use an approach that keeps the outcome continuous 11, 12, 13.

## Supporting information

Supporting info item
