# Supplementary material for: Quantifying the impact of different approaches for handling continuous predictors on the performance of a prognostic model
Source: Stat Med. 2016 May 18;35(23):4124–35. doi: 10.1002/sim.6986 (PMC5026162; doi:10.1002/sim.6986)
Supplement: Supplementary file 1 — Supporting info item [file SIM-35-4124-s001.docx]

**Other performance measures calculated**

We examined the *D* statistic, which can be interpreted as the separation between two survival curves (i.e., a difference in log hazard ratio) for two equally sized prognostic groups derived from Cox regression [1]. It is closely related to the standard deviation of the prognostic index, ${PI=\beta}_{1}x_{1}+\beta_{2}x_{2}+\cdots\beta_{k}x_{k}.$ *D* is calculated by ordering the values from the prognostic index, transforming them using expected standard normal order statistics, dividing the result by $\kappa=\sqrt{8/\pi}$ and fitting this in a single term Cox regression. *D* and its standard error are given by the coefficient and standard error in the single term Cox regression model.

We graphically examined the calibration of the models at a single time point, 10 years, using the val.surv function in the rms library in R. For each random sample, hazard regression with linear splines was used to relate the predicted probabilities from the models at 10 years to the observed event times (and censoring indicators). The actual event probability at 10 years was estimated as a function of the estimate event probability at 10 years. We investigated the influence of the approach used to handle continuous predictors on calibration by overlaying plots of observed outcomes against predicted probabilities for each of the 200 random samples.

We calculated the explained variation *R^2^* (based on the *D* statistic [1]) and the Brier score [2] as overall measures of predictive ability. *R^2^* is the proportion of the variance that is explained by the prognostic model [3, 4], defined as

$R_{D}^{2}=\frac{{D^{2}}/{\kappa^{2}}}{\sigma^{2}+{D^{2}}/{\kappa^{2}}}$

where *D* is the value of the *D* statistic,$\sigma^{2}={\pi^{2}}/6$ and $\kappa=\sqrt{8/\pi}$. The Brier score for survival data is a measure of the average discrepancy across individuals between the true disease status (0 or 1) and the predicted probability of developing the disease [5, 6], defined as

$$BS\left( t \right)=\frac{1}{n}\sum_{i=1}^{n} \left[ \frac{\left( 0-\hat{S}\left( t|X_{i},Z_{i} \right) \right)^{2}\cdot I\left( t_{i}\leq t, \delta_{i}=1 \right)}{\hat{G}\left( t_{i} \right)}+\frac{\left( 1-\hat{S}\left( t|X_{i},Z_{i} \right) \right)^{2}\cdot I\left( t_{i}>t \right)}{\hat{G}\left( t \right)} \right]$$

where $\hat{S}\left( \cdot|X_{i},Z_{i} \right)$ is the predicted probability of an event for individual *I* and $\hat{G}$ is the Kaplan-Meier estimate of the censoring distribution [5-7].

Supplementary Table 1: Degrees of freedom for each model

| **Approach** | **Model** | |
| --- | --- | --- |
|  | **CVD** | **Hip fracture** |
| Linear (all continuous) | 7 | 5 |
| Age (only) dichotomised at the median | 7 | 5 |
| Dichotomised all continuous predictors at the median | 7 | 5 |
| Dichotomised all continuous predictors at the optimal | 7 | 5 |
| Categorised into 5-year age categories | 13 | 11 |
| Categorised into 10-year age categories | 9 | 7 |
| Categorised all continuous predictors into thirds | 11 | 7 |
| Categorised all continuous predictors into fourths | 15 | 9 |
| Categorised all continuous predictors into fifths | 19 | 11 |
| Age (only) categorised into thirds | 8 | 6 |
| Age (only) categorised into fourths | 9 | 7 |
| Age (only) categorised into fifths | 10 | 8 |
| Fractional polynomials [df = 4] | 19 (max) | 11 (max) |
| Fractional polynomials of age only [df = 4] | 10 (max) | 8 (max) |
| Restricted cubic splines [3 knots] | 11 | 7 |
| Restricted cubic splines of age only [3 knots] | 8 | 6 |

Supplementary Figure 1: Continuous predictors from the THIN development data set. Models developed to predict hip fracture ] using the (orange) fractional polynomial approach with four degrees of freedom, (black) restricted cubic spline approach using three knots, (blue) dichotomising at the median predictor value approach, (green) categorising into five equally sized groups approach, and (red) linear approach

.

Supplementary Table 2: D statistic of the cardiovascular disease prognostic models (200 simulations) [mean (SD)]

|  | **25 events** | | **50 events** | | **100 events** | | **2000 events** | |
| --- | --- | --- | --- | --- | --- | --- | --- | --- |
|  | **Apparent performance** | **External validation** | **Apparent performance** | **External validation** | **Apparent performance** | **External validation** | **Apparent performance** | **External validation** |
| **Linear (all continuous)** | 2.1042  (0.3404) | 1.6505  (0.1275) | 1.9786  (0.2216) | 1.7505  (0.0660) | 1.9119  (0.1554) | 1.7923  (0.0318) | 1.8439  (0.0315) | 1.8346  (0.0052) |
| **Age (only) dichotomised at the median** | 1.7634  (0.2855) | 1.2795  (0.1221) | 1.6120  (0.2001) | 1.3643  (0.0807) | 1.5408  (0.1383) | 1.4190  (0.0360) | 1.4633  (0.0300) | 1.4635  (0.0052) |
| **Dichotomised all continuous predictors at the median** | 1.7282  (0.2702) | 1.2297  (0.1113) | 1.5557  (0.1917) | 1.3115  (0.0782) | 1.4854  (0.1347) | 1.3562  (0.0446) | 1.4117  (0.0283) | 1.3990  (0.0048) |
| **Dichotomised all continuous predictors at the optimal** | 2.2458  (0.3044) | 1.2362  (0.1528) | 1.9190  (0.1979) | 1.3470  (0.1135) | 1.7512  (0.1494) | 1.4262  (0.0595) | 1.4915  (0.0331) | 1.4656  (0.0331) |
| **Categorised into 5-year age categories** | 2.2742  (0.3267) | 1.3565  (0.4137) | 2.0191  (0.2092) | 1.5908  (0.0906) | 1.8993  (0.1523) | 1.6815  (0.0408) | 1.7850  (0.0321) | 1.7600  (0.0054) |
| **Categorised into 10-year age categories** | 2.0801  (0.3206) | 1.4631  (0.1373) | 1.9021  (0.2081) | 1.5903  (0.0707) | 1.8093  (0.1493) | 1.6492  (0.0325) | 1.7231  (0.0310) | 1.6986  (0.0049) |
| **Categorised all continuous predictors into thirds** | 2.0492  (0.2767) | 1.3306  (0.1293) | 1.8200  (0.1954) | 1.4385  (0.0722) | 1.7057  (0.1412) | 1.5035  (0.0397) | 1.5936  (0.0308) | 1.5608  (0.0055) |
| **Categorised all continuous predictors into fourths** | 2.2656  (0.3194) | 1.3566  (0.1225) | 1.9689  (0.2118) | 1.4854  (0.0795) | 1.8246  (0.1522) | 1.5705  (0.0419) | 1.6853  (0.0310) | 1.6539  (0.0065) |
| **Categorised all continuous predictors into fifths** | 2.4875  (0.3546) | 1.3241  (0.1422) | 2.0911  (0.2169) | 1.4980  (0.0776) | 1.9122  (0.1499) | 1.5991  (0.0454) | 1.7388  (0.0298) | 1.7058  (0.0077) |
| **Age (only) categorised into thirds** | 1.9331  (0.3027) | 1.4033  (0.1202) | 1.7734  (0.1934) | 1.4935  (0.0733) | 1.6937  (0.1470) | 1.5434  (0.0319) | 1.6101  (0.0308) | 1.5805  (0.0057) |
| **Age (only) categorised into fourths** | 2.0364  (0.3149) | 1.4549  (0.1206) | 1.8573  (0.1979) | 1.5527  (0.0769) | 1.7678  (0.1489) | 1.6124  (0.0334) | 1.6821  (0.0309) | 1.6552  (0.0060) |
| **Age (only) categorised into fifths** | 2.0920  (0.2986) | 1.4755  (0.1212) | 1.9069  (0.2019) | 1.5877  (0.0661) | 1.8138  (0.1459) | 1.6483  (0.0336) | 1.7232  (0.0315) | 1.7000  (0.0076) |
| **Fractional polynomials [df = 4]** | 2.1809  (0.3492) | 1.5956  (0.2366) | 2.0050  (0.2173) | 1.7073  (0.2640) | 1.9293  (0.1603) | 1.7746  (0.0406) | 1.8659  (0.0333) | 1.8525  (0.0085) |
| **Fractional polynomials of age only [df = 4]** | 2.1553  (0.3376) | 1.6238  (0.1387) | 1.9958  (0.2200) | 1.7315  (0.0689) | 1.9203  (0.1562) | 1.7781  (0.0356) | 1.8371  (0.0324) | 1.8229  (0.0090) |
| **Restricted cubic splines**  **[3 knots]** | 2.3046  (0.3391) | 1.5246  (0.1734) | 2.0716  (0.2291) | 1.6892  (0.0877) | 1.9656  (0.1646) | 1.7683  (0.0464) | 1.8622  (0.0323) | 1.8498  (0.0069) |
| **Restricted cubic splines of age only [3 knots]** | 2.1640  (0.3313) | 1.5818  (0.1607) | 2.0019  (0.2221) | 1.7104  (0.0772) | 1.9175  (0.1573) | 1.7653  (0.0426) | 1.8378  (0.0321) | 1.8246  (0.0070) |

Supplementary Table 3: R^2^ of the cardiovascular disease prognostic models (200 simulations) [mean (SD)]

|  | **25 events** | | **50 events** | | **100 events** | | **2000 events** | |
| --- | --- | --- | --- | --- | --- | --- | --- | --- |
|  | **Apparent performance** | **External validation** | **Apparent performance** | **External validation** | **Apparent performance** | **External validation** | **Apparent performance** | **External validation** |
| **Linear (all continuous)** | 0.5139  (0.0767) | 0.3941  (0.0377) | 0.4831  (0.0553) | 0.4225  (0.0188) | 0.4660  (0.04030 | 0.4340  (0.0088) | 0.4480  (0.0085) | 0.4455  (0.0014) |
| **Age (only) dichotomised at the median** | 0.4218  (0776) | 0.2808  (0.0385) | 0.3810  (0.0580) | 0.3075  (0.0253) | 0.3609  (0.0411) | 0.3246  (0.0112) | 0.3382  (0.0092) | 0.3383  (0.0016) |
| **Dichotomised all continuous predictors at the median** | 0.4125  (0.0748) | 0.2652  (0.0351) | 0.3646  (0.0563) | 0.2910  (0.0247) | 0.3443  (0.0405) | 0.3051  (0.0140) | 0.3224  (0.0088) | 0.3184  (0.0015) |
| **Dichotomised all continuous predictors at the optimal** | 0.5410  (0.0666) | 0.2673  (0.0479) | 0.4656  (0.0512) | 0.3020  (0.0356) | 0.4215  (0.0412) | 0.3268  (0.0184) | 0.3468  (0.0100) | 0.3390  (0.0071) |
| **Categorised into 5-year age categories** | 0.5466  (0.0700) | 0.3209  (0.0498) | 0.4907  (0.0517) | 0.3763  (0.0272) | 0.4614  (0.0396) | 0.4029  (0.0117) | 0.4320  (0.0088) | 0.4251  (0.0015) |
| **Categorised into 10-year age categories** | 0.5022  (0.0761) | 0.3376  (0.0422) | 0.4610  (0.0544) | 0.3762  (0.0212) | 0.4374  (0.0403) | 0.3936  (0.0094) | 0.4147  (0.0087) | 0.4079  (0.0014) |
| **Categorised all continuous predictors into thirds** | 0.4962  (0.0679) | 0.2968  (0.0405) | 0.4395  (0.0526) | 0.3305  (0.0224) | 0.4088  (0.0398) | 0.3504  (0.0121) | 0.3774  (0.0091) | 0.3677  (0.0017) |
| **Categorised all continuous predictors into fourths** | 0.5449  (0.0696) | 0.3049  (0.0383) | 0.4780  (0.0529) | 0.3448  (0.0244) | 0.4415  (0.0408) | 0.3705  (0.0125) | 0.4040  (0.0089) | 0.3951  (0.0019) |
| **Categorised all continuous predictors into fifths** | 0.5897  (0.0682) | 0.2947  (0.0444) | 0.5080  (0.0518) | 0.3486  (0.0236) | 0.4648  (0.0388) | 0.3790  (0.0135) | 0.4192  (0.0084) | 0.4099  (0.0022) |
| **Age (only) categorised into thirds** | 0.4664  (0.0769) | 0.3194  (0.0373) | 0.4268  (0.0533) | 0.3473  (0.0225) | 0.4053  (0.0415) | 0.3625  (0.0096) | 0.3822  (0.0090) | 0.3736  (0.0017) |
| **Age (only) categorised into fourths** | 0.4918  (0.0761) | 0.3352  (0.0372) | 0.4494  (0.0526) | 0.3651  (0.0233) | 0.4261  (0.0408) | 0.3829  (0.0098) | 0.4031  (0.0088) | 0.3954  (0.0017) |
| **Age (only) categorised into fifths** | 0.5059  (0.0697) | 0.3415  (0.0371) | 0.4623  (0.0524) | 0.3755  (0.0198) | 0.4387  (0.0393) | 0.3934  (0.0098) | 0.4148  (0.0089) | 0.4083  (0.0022) |
| **Fractional polynomials [df = 4]** | 0.5249  (0.0773) | 0.3799  (0.0467) | 0.4870  (0.0540) | 0.4153  (0.0209) | 0.4690  (0.0412) | 0.4291  (0.0113) | 0.4538  (0.0089) | 0.4503  (0.0023) |
| **Fractional polynomials of age only [df = 4]** | 0.5194  (0.0757) | 0.3855  (0.0413) | 0.4846  (0.0548) | 0.4169  (0.0197) | 0.4668  (0.0404) | 0.4301  (0.0099) | 0.4461  (0.0087) | 0.4424  (0.0024) |
| **Restricted cubic splines**  **[3 knots]** | 0.5527  (0.0712) | 0.3559  (0.0525) | 0.5030  (0.0553) | 0.4048  (0.0254) | 0.4782  (0.0417) | 0.4273  (0.0130) | 0.4529  (0.0087) | 0.4496  (0.0019) |
| **Restricted cubic splines of age only [3 knots]** | 0.5216  (0.0740) | 0.3730  (0.0483) | 0.4861  (0.0551) | 0.4109  (0.0222) | 0.4660  (0.0407) | 0.4265  (0.0120) | 0.4463  (0.0086) | 0.4428  (0.0019) |

Supplementary Table 4: Brier score of the cardiovascular disease prognostic models (200 simulations) [mean (SD)]

|  | **25 events** | | **50 events** | | **100 events** | | **2000 events** | |
| --- | --- | --- | --- | --- | --- | --- | --- | --- |
|  | **Apparent performance** | **External validation** | **Apparent performance** | **External validation** | **Apparent performance** | **External validation** | **Apparent performance** | **External validation** |
| **Linear (all continuous)** | 0.0595  (0.0080) | 0.0642  (0.0030) | 0.0601  (0.0058) | 0.0626  (0.0013) | 0.0601  (0.0043) | 0.0620  (0.0008) | 0.0618  (0.0009) | 0.0613  (0.0001) |
| **Age (only) dichotomised at the median** | 0.0631  (0.0068) | 0.0683  (0.0032) | 0.0649  (0.0057) | 0.0644  (0.0015) | 0.0647  (0.0042) | 0.0656  (0.0010) | 0.0657  (0.0008) | 0.0648  (0.0002) |
| **Dichotomised all continuous predictors at the median** | 0.0635  (0.0069) | 0.0679  (0.0029) | 0.0654  (0.0056) | 0.0663  (0.0013) | 0.0650  (0.0041) | 0.0656  (0.0008) | 0.0659  (0.0008) | 0.0649  (0.0001) |
| **Dichotomised all continuous predictors at the optimal** | 0.0566  (0.0081) | 0.0700  (0.0038) | 0.0605  (0.0057) | 0.0671  (0.0020) | 0.0619  (0.0043) | 0.0655  (0.0011) | 0.0653  (0.0008) | 0.0646  (0.0002) |
| **Categorised into 5-year age categories** | 0.0571  (0.0077) | 0.0680  (0.0032) | 0.0599  (0.0057) | 0.0647  (0.0017) | 0.0609  (0.0043) | 0.0633  (0.0009) | 0.0624  (0.0009) | 0.0620  (0.0001) |
| **Categorised into 10-year age categories** | 0.0592  (0.0077) | 0.0672  (0.0035) | 0.0612  (0.0057) | 0.0647  (0.0015) | 0.0619  (0.0043) | 0.0636  (0.0009) | 0.0630  (0.0008) | 0.0627  (0.0001) |
| **Categorised all continuous predictors into thirds** | 0.0600  (0.0073) | 0.0686  (0.0035) | 0.0628  (0.0056) | 0.0661  (0.0015) | 0.0631  (0.0040) | 0.0649  (0.0090) | 0.0644  (0.0008) | 0.0639  (0.0001) |
| **Categorised all continuous predictors into fourths** | 0.0578  (0.0075) | 0.0698  (0.0037) | 0.0613  (0.0055) | 0.0664  (0.0019) | 0.0622  (0.0042) | 0.0647  (0.0010) | 0.0638  (0.0009) | 0.0633  (0.0001) |
| **Categorised all continuous predictors into fifths** | 0.0556  (0.0072) | 0.0718  (0.0052) | 0.0598  (0.0057) | 0.0667  (0.0019) | 0.0612  (0.0042) | 0.0646  (0.0011) | 0.0632  (0.0008) | 0.0628  (0.0002) |
| **Age (only) categorised into thirds** | 0.0612  (0.0072) | 0.0676  (0.0034) | 0.0631  (0.0055) | 0.0656  (0.0015) | 0.0633  (0.0042) | 0.0647  (0.0009) | 0.0643  (0.0008) | 0.0639  (0.0002) |
| **Age (only) categorised into fourths** | 0.0601  (0.0072) | 0.0674  (0.0037) | 0.0623  (0.0055) | 0.0652  (0.0015) | 0.0626  (0.0043) | 0.0642  (0.0009) | 0.0638  (0.0008) | 0.0633  (0.0001) |
| **Age (only) categorised into fifths** | 0.0595  (0.0072) | 0.0671  (0.0036) | 0.0616  (0.0057) | 0.0648  (0.0014) | 0.0621  (0.0043) | 0.0637  (0.0009) | 0.0633  (0.0008) | 0.0628  (0.0002) |
| **Fractional polynomials [df = 4]** | 0.0581  (0.0080) | 0.0653  (0.0034) | 0.0602  (0.0057) | 0.0631  (0.0013) | 0.0606  (0.0043) | 0.0623  (0.0009) | 0.0618  (0.0009) | 0.0613  (0.0001) |
| **Fractional polynomials of age only [df = 4]** | 0.0584  (0.0080) | 0.0650  (0.0032) | 0.0603  (0.0058) | 0.0630  (0.0013) | 0.0607  (0.0043) | 0.0622  (0.0008) | 0.0619  (0.0008) | 0.0614  (0.0001) |
| **Restricted cubic splines**  **[3 knots]** | 0.0570  (0.0077) | 0.0677  (0.0057) | 0.0598  (0.0058) | 0.0640  (0.0017) | 0.0605  (0.0043) | 0.0626  (0.0010) | 0.0618  (0.0009) | 0.0613  (0.0001) |
| **Restricted cubic splines of age only [3 knots]** | 0.0584  (0.0077) | 0.0661  (0.0048) | 0.0604  (0.0057) | 0.0633  (0.0014) | 0.0608  (0.0043) | 0.0624  (0.0009) | 0.0619  (0.0008) | 0.0614  (0.0001) |

**Supplementary Figure 2: External validation calibration plots of cardiovascular disease risk (25 events)**


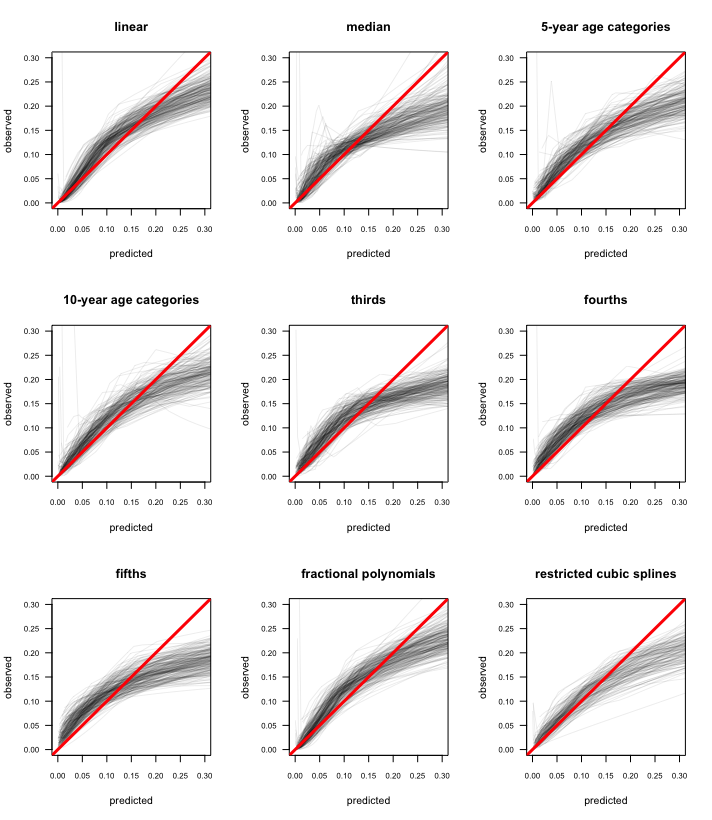


**Supplementary Figure 3: External validation calibration plots of cardiovascular disease risk (50 events)**


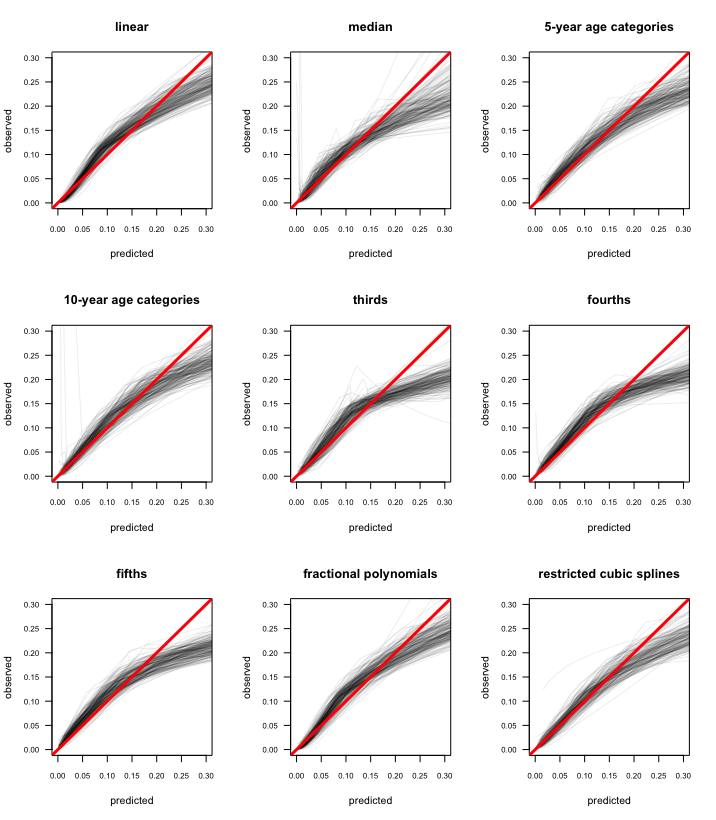


**Supplementary Figure 4: External validation calibration plots of cardiovascular disease risk (100 events)**


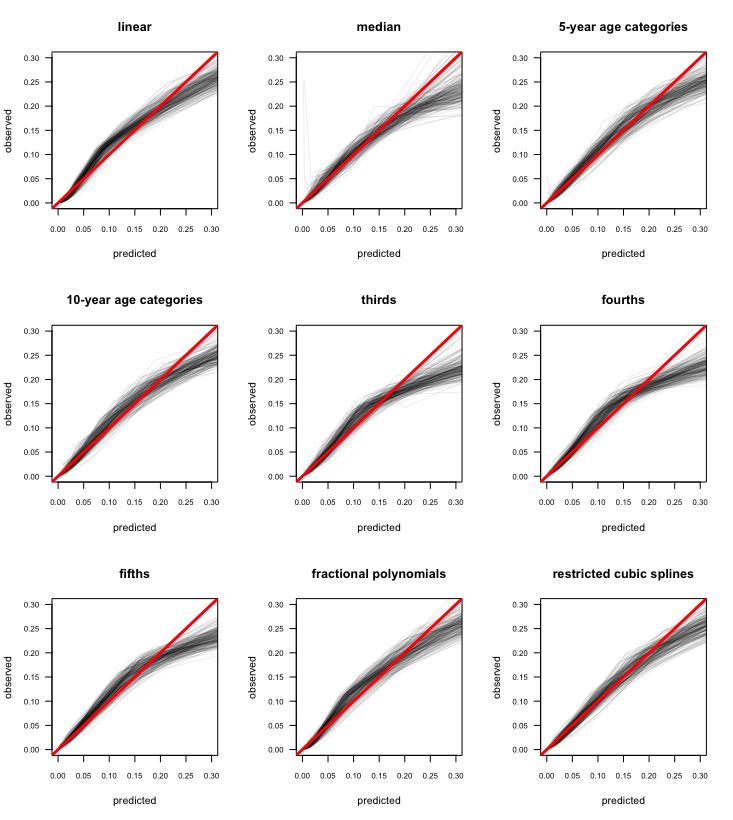


Supplementary Table 5: D statistic of the hip fracture prognostic models (200 simulations) [mean (SD)]

|  | **25 events** | | **50 events** | | **100 events** | | **2000 events** | |
| --- | --- | --- | --- | --- | --- | --- | --- | --- |
|  | **Apparent performance** | **External validation** | **Apparent performance** | **External validation** | **Apparent performance** | **External validation** | **Apparent performance** | **External validation** |
| **Linear (all continuous)** | 2.8445  (0.3228) | 2.4091  (0.2361) | 2.7742  (0.2329) | 2.5322  (0.0950) | 2.7335  (0.1591) | 2.5611  (0.0332) | 2.6770  (0.0337) | 2.5782  (0.0113) |
| **Age (only) dichotomised at the median** | 1.8475  (0.2545) | 1.5760  (0.2001) | 1.7854  (0.1908) | 1.6694  (0.0755) | 1.7438  (0.1466) | 1.6998  (0.0460) | 1.7193  (0.0284) | 1.7392  (0.0079) |
| **Dichotomised all continuous predictors at the median** | 1.8681  (0.2549) | 1.5705  (0.1817) | 1.7642  (0.1776) | 1.6469  (0.0923) | 1.7161  (0.1407) | 1.6784  (0.0503) | 1.6709  (0.0290) | 1.7120  (0.0032) |
| **Dichotomised all continuous predictors at the optimal** | 2.5341  (0.3229) | 1.7934  (0.4062) | 2.3337  (0.2221) | 1.9186  (0.1288) | 2.2036  (0.1585) | 1.9452  (0.0977) | 1.6284  (0.0338) | 1.6459  (0.0186) |
| **Categorised into 5-year age categories** | 2.6615  (0.2715) | 2.2006  (0.1484) | 2.5636  (0.2061) | 2.3148  (0.0853) | 2.5029  (0.1380) | 2.3783  (0.0484) | 2.4562  (0.0312) | 2.45400  (0.0104) |
| **Categorised into 10-year age categories** | 2.4759  (0.2822) | 2.1198  (0.1663) | 2.4016  (0.2025) | 2.2213  (0.0767) | 2.3502  (0.1414) | 2.2627  (0.0372) | 2.3185  (0.0298) | 2.3078  (0.0074) |
| **Categorised all continuous predictors into thirds** | 2.2786  (0.2933) | 1.9203  (0.1515) | 2.1816  (0.2031) | 2.0065  (0.0849) | 2.1109  (0.1504) | 2.0517  (0.0617) | 2.0302  (0.0284) | 2.0474  (0.0102) |
| **Categorised all continuous predictors into fourths** | 2.4917  (0.2846) | 2.0437  (0.1462) | 2.3717  (0.2004) | 2.1344  (0.0826) | 2.3070  (0.1449) | 2.1788  (0.0566) | 2.2421  (0.0288) | 2.2192  (0.0095) |
| **Categorised all continuous predictors into fifths** | 2.6561  (0.2999) | 2.1233  (0.1356) | 2.4982  (0.2063) | 2.2309  (0.0716) | 2.4203  (0.1383) | 2.2862  (0.0498) | 2.3598  (0.0305) | 2.3328  (0.0088) |
| **Age (only) categorised into thirds** | 2.2090  (0.2602) | 1.9122  (0.1650) | 2.1570  (0.1915) | 2.0020  (0.0749) | 2.1052  (0.1433) | 2.0425  (0.0432) | 2.0495  (0.0278) | 2.0405  (0.0074) |
| **Age (only) categorised into fourths** | 2.3954  (0.2660) | 2.0519  (0.1573) | 2.3204  (0.1971) | 2.1377  (0.0681) | 2.2756  (0.1400) | 2.1703  (0.0438) | 2.2384  (0.0287) | 2.2101  (0.0082) |
| **Age (only) categorised into fifths** | 2.5145  (0.2797) | 2.1624  (0.1421) | 2.4278  (0.2019) | 2.2523  (0.0636) | 2.3769  (0.1367) | 2.2906  (0.0377) | 2.3451  (0.0299) | 2.3350  (0.0091) |
| **Fractional polynomials [df = 4]** | 2.8551  (0.3239) | 2.3985  (0.2418) | 2.7825  (0.2339) | 2.5295  (0.0972) | 2.7416  (0.1632) | 2.5554  (0.0492) | 2.6819  (0.0347) | 2.6039  (0.0111) |
| **Fractional polynomials of age only [df = 4]** | 2.8454  (0.3222) | 2.4083  (0.2358) | 2.7742  (0.2329) | 2.5321  (0.0951) | 2.7334  (0.1593) | 2.5606  (0.0337) | 2.6761  (0.0339) | 2.5776  (0.0111) |
| **Restricted cubic splines**  **[3 knots]** | 2.8772  (0.3365) | 2.3879  (0.2286) | 2.7969  (0.2322) | 2.5260  (0.0939) | 2.7411  (0.1597) | 2.5626  (0.0316) | 2.6786  (0.0337) | 2.5916  (0.0120) |
| **Restricted cubic splines of age only [3 knots]** | 2.8472  (0.3245) | 2.4131  (0.2215) | 2.7759  (0.2323) | 2.5316  (0.0946) | 2.7329  (0.1586) | 2.5606  (0.0334) | 2.6769  (0.0337) | 2.5782  (0.0114) |

Supplementary Table 6: R^2^ of the hip fracture prognostic models (200 simulations) [mean (SD)]

|  | **25 events** | | **50 events** | | **100 events** | | **2000 events** | |
| --- | --- | --- | --- | --- | --- | --- | --- | --- |
|  | **Apparent performance** | **External validation** | **Apparent performance** | **External validation** | **Apparent performance** | **External validation** | **Apparent performance** | **External validation** |
| **Linear (all continuous)** | 0.6542  (0.0513) | 0.5776  (0.0535) | 0.6450  (0.0384) | 0.6044  (0.0203) | 0.6396  (0.0270) | 0.6102  (0.0062) | 0.6311  (0.0059) | 0.6134  (0.0021) |
| **Age (only) dichotomised at the median** | 0.4454  (0.0676) | 0.3711  (0.0607) | 0.4301  (0.0520) | 0.3992  (0.0221) | 0.4195  (0.0409) | 0.4081  (0.0132) | 0.4137  (0.0080) | 0.4193  (0.0022) |
| **Dichotomised all continuous predictors at the median** | 0.4508  (0.0669) | 0.3695  (0.0550) | 0.4246  (0.0483) | 0.3926  (0.0274) | 0.4118  (0.0396) | 0.4020  (0.0145) | 0.3999  (0.0083) | 0.4117  (0.0009) |
| **Dichotomised all continuous predictors at the optimal** | 0.5999  (0.0593) | 0.4400  (0.0655) | 0.5625  (0.0463) | 0.4668  (0.0345) | 0.5354  (0.0356) | 0.4741  (0.0250) | 0.3876  (0.0099) | 0.3927  (0.0054) |
| **Categorised into 5-year age categories** | 0.6248  (0.0477) | 0.5349  (0.0350) | 0.6086  (0.0377) | 0.5608  (0.0189) | 0.5983  (0.0266) | 0.5744  (0.0100) | 0.5902  (0.0062) | 0.5898  (0.0021) |
| **Categorised into 10-year age categories** | 0.5898  (0.0549) | 0.5160  (0.0412) | 0.5770  (0.0410) | 0.5405  (0.0179) | 0.5676  (0.0295) | 0.5499  (0.0082) | 0.5620  (0.0063) | 0.5595  (0.0016) |
| **Categorised all continuous predictors into thirds** | 0.5487  (0.0610) | 0.4669  (0.0405) | 0.5295  (0.0452) | 0.4987  (0.0215) | 0.5141  (0.0353) | 0.5010  (0.0152) | 0.4959  (0.0070) | 0.5002  (0.0025) |
| **Categorised all continuous predictors into fourths** | 0.5929  (0.0538) | 0.4980  (0.0372) | 0.5710  (0.0410) | 0.5206  (0.0197) | 0.5584  (0.0307) | 0.5311  (0.0130) | 0.5454  (0.0064) | 0.5404  (0.0021) |
| **Categorised all continuous predictors into fifths** | 0.6231  (0.0525) | 0.5173  (0.0331) | 0.5961  (0.0396) | 0.5427  (0.0162) | 0.5820  (0.0277) | 0.5550  (0.0108) | 0.5707  (0.0063) | 0.5650  (0.0019) |
| **Age (only) categorised into thirds** | 0.5342  (0.0590) | 0.4646  (0.0457) | 0.5241  (0.0443) | 0.4886  (0.0193) | 0.5129  (0.0339) | 0.4989  (0.0107) | 0.5007  (0.0068) | 0.4985  (0.0018) |
| **Age (only) categorised into fourths** | 0.5742  (0.0538) | 0.4998  (0.0408) | 0.5603  (0.0417) | 0.5215  (0.0163) | 0.5517  (0.0303) | 0.5292  (0.0101) | 0.5446  (0.0064) | 0.5383  (0.0018) |
| **Age (only) categorised into fifths** | 0.5974  (0.0532) | 0.5263  (0.0347) | 0.5824  (0.0402) | 0.5475  (0.0143) | 0.5732  (0.0281) | 0.5560  (0.0082) | 0.5676  (0.0063) | 0.5655  (0.0019) |
| **Fractional polynomials [df = 4]** | 0.6559  (0.0513) | 0.5753  (0.0549) | 0.6464  (0.0385) | 0.6038  (0.0207) | 0.6409  (0.0276) | 0.6091  (0.0096) | 0.6319  (0.0060) | 0.6181  (0.0020) |
| **Fractional polynomials of age only [df = 4]** | 0.6544  (0.0512) | 0.5775  (0.0534) | 0.6450  (0.0384) | 0.6043  (0.0203) | 0.6395  (0.0270) | 0.6101  (0.0063) | 0.6309  (0.0059) | 0.6133  (0.0020) |
| **Restricted cubic splines**  **[3 knots]** | 0.6591  (0.0522) | 0.5735  (0.0520) | 0.6488  (0.0377) | 0.6032  (0.0200) | 0.6408  (0.0269) | 0.6105  (0.0059) | 0.6313  (0.0059) | 0.6159  (0.0022) |
| **Restricted cubic splines of age only [3 knots]** | 0.6546  (0.0513) | 0.5788  (0.0499) | 0.6453  (0.0383) | 0.6042  (0.0201) | 0.6395  (0.0269) | 0.6101  (0.0062) | 0.6310  (0.0059) | 0.6134  (0.0021) |

Supplementary Table 7: Brier score of the hip fracture prognostic models (200 simulations) [mean (SD)]

|  | **25 events** | | **50 events** | | **100 events** | | **2000 events** | |
| --- | --- | --- | --- | --- | --- | --- | --- | --- |
|  | **Apparent performance** | **External validation** | **Apparent performance** | **External validation** | **Apparent performance** | **External validation** | **Apparent performance** | **External validation** |
| **Linear (all continuous)** | 0.0129  (0.0017) | 0.0149  (0.0002) | 0.0129  (0.0010) | 0.0148  (0.00011) | 0.0129  (0.0009) | 0.0148  (0.00009) | 0.0130  (0.00002) | 0.0147  (0.00002) |
| **Age (only) dichotomised at the median** | 0.0140  (0.0018) | 0.0158  (0.0001) | 0.0139  (0.0011) | 0.0157  (0.00004) | 0.0139  (0.0009) | 0.0157  (0.00003) | 0.0140  (0.00002) | 0.0157  (0.00001) |
| **Dichotomised all continuous predictors at the median** | 0.0141  (0.0018) | 0.0158  (0.0001) | 0.0139  (0.0011) | 0.0157  (0.00004) | 0.0140  (0.0009) | 0.0157  (0.00003) | 0.0140  (0.00002) | 0.0157  (0.00001) |
| **Dichotomised all continuous predictors at the optimal** | 0.0130  (0.0017) | 0.0153  (0.0002) | 0.0130  (0.0011) | 0.0151  (0.00010) | 0.0132  (0.0009) | 0.0151  (0.00008) | 0.0140  (0.00021) | 0.0157  (0.00001) |
| **Categorised into 5-year age categories** | 0.0132  (0.0017) | 0.0152  (0.0002) | 0.0132  (0.0011) | 0.0151  (0.00008) | 0.0133  (0.0009) | 0.0150  (0.00006) | 0.0134  (0.00002) | 0.0150  (0.00001) |
| **Categorised into 10-year age categories** | 0.0135  (0.0017) | 0.0154  (0.0002) | 0.0134  (0.0011) | 0.0153  (0.00006) | 0.0135  (0.0009) | 0.0152  (0.00004) | 0.0136  (0.00002) | 0.0152  (0.00001) |
| **Categorised all continuous predictors into thirds** | 0.0138  (0.0018) | 0.0156  (0.0001) | 0.0137  (0.0011) | 0.0155  (0.00005) | 0.0137  (0.0009) | 0.0155  (0.00003) | 0.0138  (0.00002) | 0.0155  (0.00001) |
| **Categorised all continuous predictors into fourths** | 0.0136  (0.0018) | 0.0155  (0.0002) | 0.0135  (0.0011) | 0.0154  (0.00006) | 0.0136  (0.0009) | 0.0154  (0.00004) | 0.0137  (0.00002) | 0.0153  (0.00001) |
| **Categorised all continuous predictors into fifths** | 0.0133  (0.0017) | 0.0154  (0.0002) | 0.0133  (0.0011) | 0.0153  (0.00007) | 0.0134  (0.0009) | 0.0152  (0.00005) | 0.0135  (0.00002) | 0.0152  (0.00001) |
| **Age (only) categorised into thirds** | 0.0138  (0.0018) | 0.0156  (0.0001) | 0.0137  (0.0011) | 0.0155  (0.00005) | 0.0137  (0.0009) | 0.0155  (0.00003) | 0.0138  (0.00002) | 0.0155  (0.00001) |
| **Age (only) categorised into fourths** | 0.0136  (0.0018) | 0.0155  (0.0001) | 0.0135  (0.0011) | 0.0154  (0.00006) | 0.0136  (0.0009) | 0.0153  (0.00004) | 0.0137  (0.00002) | 0.0153  (0.00001) |
| **Age (only) categorised into fifths** | 0.0135  (0.0017) | 0.0153  (0.0002) | 0.0134  (0.0011) | 0.0152  (0.00007) | 0.0134  (0.0009) | 0.0152  (0.00005) | 0.0135  (0.00002) | 0.0152  (0.00001) |
| **Fractional polynomials [df = 4]** | 0.0129  (0.0017) | 0.0149  (0.0002) | 0.0128  (0.0010) | 0.0148  (0.00012) | 0.0129  (0.0009) | 0.0148  (0.00010) | 0.0130  (0.0002) | 0.0147  (0.00003) |
| **Fractional polynomials of age only [df = 4]** | 0.0129  (0.0017) | 0.0149  (0.0002) | 0.0129  (0.0010) | 0.0148  (0.00011) | 0.0129  (0.0009) | 0.0148  (0.00009) | 0.0130  (0.0002) | 0.0147  (0.00002) |
| **Restricted cubic splines**  **[3 knots]** | 0.0129  (0.0017) | 0.0168  (0.0085) | 0.0128  (0.0010) | 0.0148  (0.00030) | 0.0129  (0.0009) | 0.0147  (0.00013) | 0.0130  (0.00002) | 0.0147  (0.00003) |
| **Restricted cubic splines of age only [3 knots]** | 0.0129  (0.0017) | 0.0168  (0.0085) | 0.0129  (0.0010) | 0.0148  (0.00028) | 0.0129  (0.0009) | 0.0147  (0.00012) | 0.0130  (0.00002) | 0.0147  (0.00003) |

**Supplementary Figure 5: External validation calibration plots of hip fracture risk (25 events)**


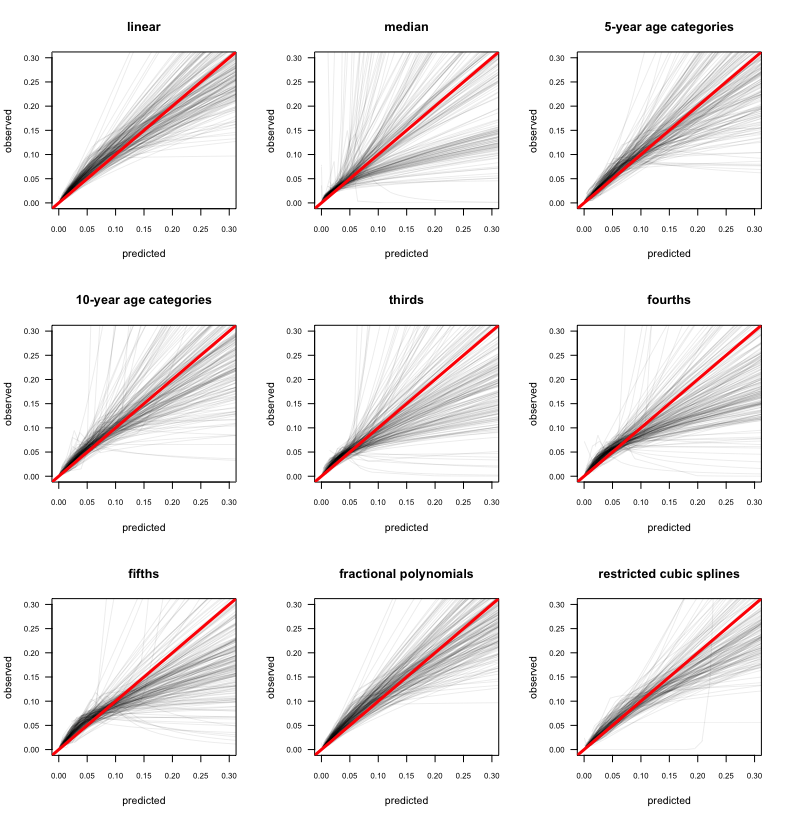


**Supplementary Figure 6: External validation calibration plots of hip fracture risk (50 events)**


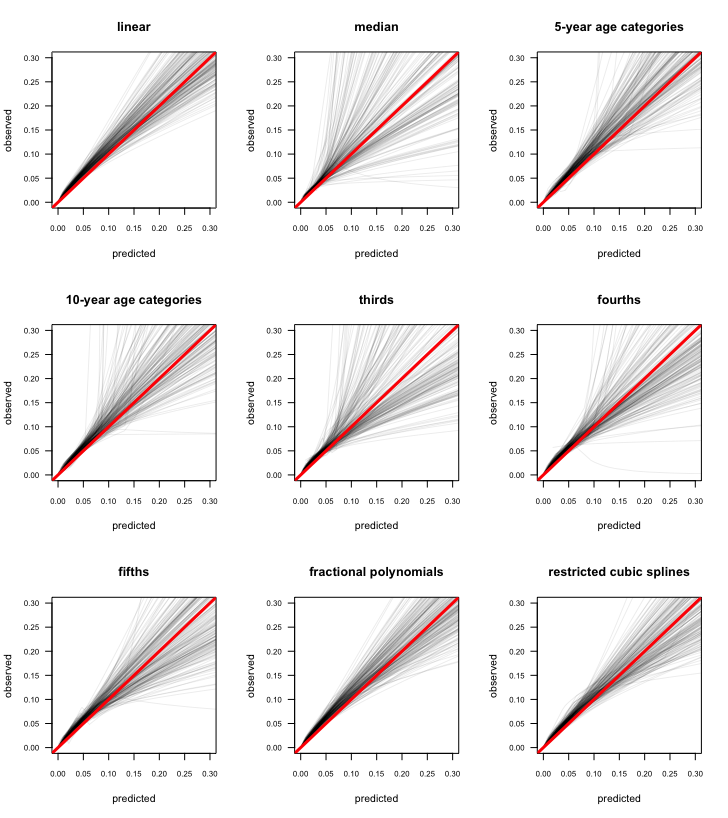


**Supplementary Figure 7: External validation calibration plots of hip fracture risk (100 events)**


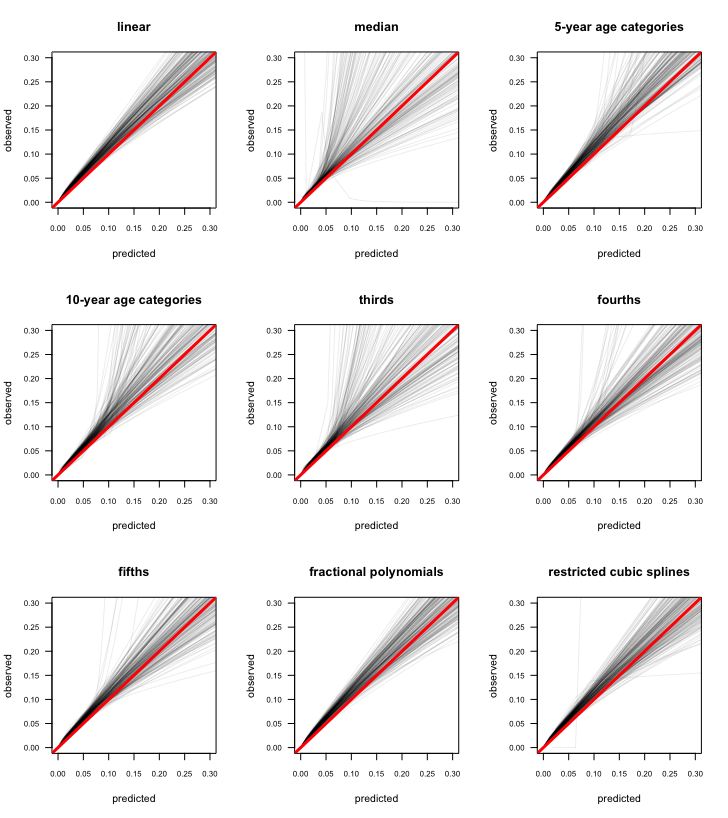


**Supplementary Figure 8: External validation calibration plots of hip fracture risk (2000 events)**


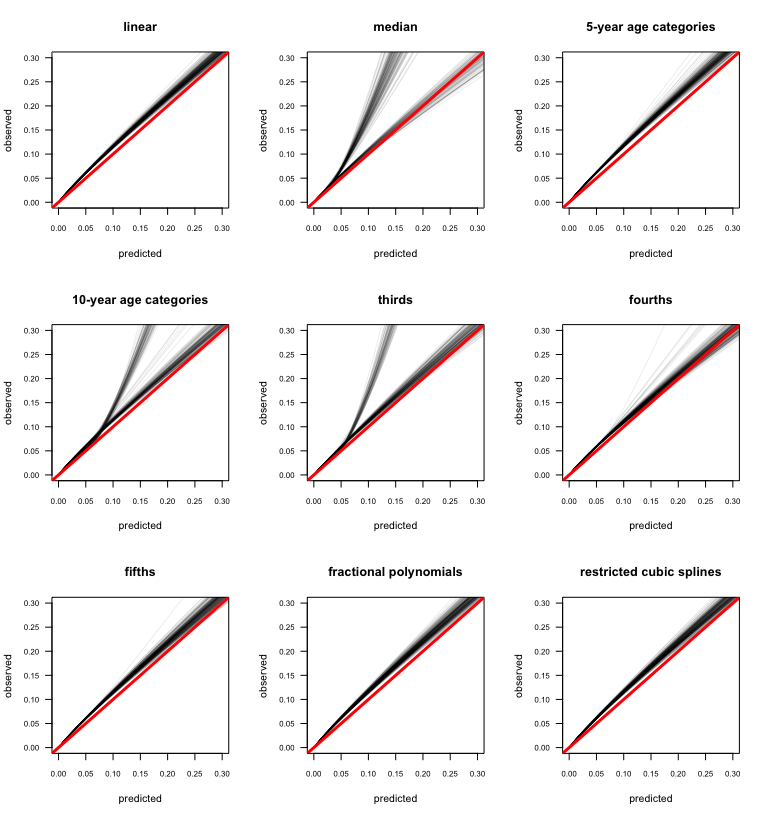


**Supplementary Figure 9: Net benefit of using decision curve analysis when predicting cardiovascular disease (2,000 events)**

**REFERENCES**

1. Royston P, Sauerbrei W. A new measure of prognostic separation in survival data. *Stat Med* 2004; **23**: 723-748.

2. Steyerberg EW, Vickers AJ, Cook NR, Gerds T, Gonen M, Obuchowski N, Pencina MJ, Kattan MW. Assessing the performance of prediction models: a framework for traditional and novel measures. *Epidemiology* 2010; **21**: 128-138.

3. Royston P. Explained variation for survival models. *Stata Journal* 2006; **6**: 83-96.

4. Schemper M. Predictive accuracy and explained variation. *Stat Med* 2003; **22**: 2299-2308.

5. Schumacher M, Binder H, Gerds T. Assessment of survival prediction models based on microarray data. *Bioinformatics* 2007; **23**: 1768-1774.

6. Graf E, Schmoor C, Sauerbrei W, Schumacher M. Assessment and comparison of prognostic classification schemes for survival data. *Stat Med* 1999; **18**: 2529-2545.

7. Gerds T, Schumacher M. Consistent estimation of the expected Brier score in general survival models with right-censored event times. *Biom J* 2006; **6**: 1029-1040.
